# Neural Processes of Psychological Stress and Relaxation Predict the Future Evolution of Quality of Life in Multiple Sclerosis

**DOI:** 10.3389/fneur.2021.753107

**Published:** 2021-11-23

**Authors:** Lil Meyer-Arndt, Tanja Schmitz-Hübsch, Judith Bellmann-Strobl, Alexander U. Brandt, John-Dylan Haynes, Stefan M. Gold, Friedemann Paul, Martin Weygandt

**Affiliations:** ^1^Max Delbrück Center for Molecular Medicine and Charité — Universitätsmedizin Berlin, Corporate Member of Freie Universität Berlin, Humboldt-Universität zu Berlin, and Berlin Institute of Health, Experimental and Clinical Research Center, Berlin, Germany; ^2^Charité - Universitätsmedizin Berlin, Corporate Member of Freie Universität Berlin, Humboldt-Universität zu Berlin, and Berlin Institute of Health, NeuroCure Clinical Research Center, Berlin, Germany; ^3^Charité - Universitätsmedizin Berlin, Corporate Member of Freie Universität Berlin, Humboldt-Universität zu Berlin, and Berlin Institute of Health, Department of Neurology, Berlin, Germany; ^4^Department of Neurology, University of California, Irvine, Irvine, CA, United States; ^5^Charité - Universitätsmedizin Berlin, Corporate Member of Freie Universität Berlin, Humboldt-Universität zu Berlin, and Berlin Institute of Health, Berlin Center for Advanced Neuroimaging, Berlin, Germany; ^6^Charité - Universitätsmedizin Berlin, Corporate Member of Freie Universität Berlin, Humboldt-Universität zu Berlin, Berlin Institute of Health, Bernstein Center for Computational Neuroscience, Berlin, Germany; ^7^Charité - Universitätsmedizin Berlin, Corporate Member of Freie Universität Berlin, Humboldt-Universität zu Berlin, and Berlin Institute of Health, Department of Psychosomatic Medicine, Berlin, Germany; ^8^Charité - Universitätsmedizin Berlin, Corporate Member of Freie Universität Berlin, Humboldt-Universität zu Berlin, and Berlin Institute of Health, Department of Psychiatry and Psychotherapy, Campus Benjamin Franklin, Berlin, Germany; ^9^Universitätsklinikum Hamburg-Eppendorf, Institute of Neuroimmunology and Multiple Sclerosis (INIMS), Center for Molecular Neurobiology Hamburg, Hamburg, Germany

**Keywords:** multiple sclerosis, quality of life, neuropsychiatric symptoms, psychological stress, functional magnet resonance imaging (fMRI)

## Abstract

Health-related quality of life (HRQoL) is an essential complementary parameter in the assessment of disease burden and treatment outcome in multiple sclerosis (MS) and can be affected by neuropsychiatric symptoms, which in turn are sensitive to psychological stress. However, until now, the impact of neurobiological stress and relaxation on HRQoL in MS has not been investigated. We thus evaluated whether the activity of neural networks triggered by mild psychological stress (elicited in an fMRI task comprising mental arithmetic with feedback) or by stress termination (i.e., relaxation) at baseline (T0) predicts HRQoL variations occurring between T0 and a follow-up visit (T1) in 28 patients using a robust regression and permutation testing. The median delay between T0 and T1 was 902 (range: 363–1,169) days. We assessed HRQoL based on the Hamburg Quality of Life Questionnaire in MS (HAQUAMS) and accounted for the impact of established HRQoL predictors and the cognitive performance of the participants. Relaxation-triggered activity of a widespread neural network predicted future variations in overall HRQoL (*t* = 3.68, *p*_family−wise error [FWE]_-corrected = 0.008). Complementary analyses showed that relaxation-triggered activity of the same network at baseline was associated with variations in the HAQUAMS mood subscale on an α_FWE_ = 0.1 level (*t* = 3.37, *p*_FWE_ = 0.087). Finally, stress-induced activity of a prefronto-limbic network predicted future variations in the HAQUAMS lower limb mobility subscale (*t* = −3.62, *p*_FWE_ = 0.020). Functional neural network measures of psychological stress and relaxation contain prognostic information for future HRQoL evolution in MS independent of clinical predictors.

## Introduction

Multiple sclerosis (MS) is a chronic autoimmune disease of the central nervous system driven by inflammation, demyelination, and neurodegeneration (1). While sensorimotor and visual symptoms have long been considered the major disease burden of MS, it is now widely accepted that they reflect just one of the several groups of debilitating symptoms (2, 3). Additional symptoms comprise cognitive impairment (4, 5), fatigue (6), and neuropsychiatric symptoms, such as depression and anxiety (7), which together contribute to a reduced quality of life (QoL). In line with this broad range of contributing factors, QoL is a multidimensional concept that is defined as a person's subjective overall well-being and ability to participate in and enjoy life according to individual goals and expectations (8, 9). More specifically, the term health-related QoL (HRQoL) refers to QoL of an individual affected by health problems, medical conditions, and their treatments (10). Consistently, HRQoL is understood as an important complementary parameter in the assessment of MS symptoms, which otherwise may be overlooked, and a relevant patient-reported outcome for treatment success [e.g., (2, 11)].

Today, only few prognostic markers exist for MS symptom progression in general and HRQoL in particular. Male sex and younger age at onset are the only clinico-demographic factors influencing the progression to severe disability (12). Furthermore, MS studies on psychobiological stress found that (i) exposure to mild and extreme stress is associated with an increased risk for MS relapses and disease exacerbation (13, 14), (ii) participation in stress management interventions can reduce the formation of new MRI lesions (15) and (iii) hypothalamo–pituitary–adrenal (HPA) axis hyperactivity is associated with future disability accrual (16, 17). Finally, predictors of future HRQoL evolution were identified as disease duration and clinical disability (18).

Interestingly, we recently showed that neural processing of stressful stimuli is a predictor for future MS disease severity (19), whereas others found that psychobiological stress contributes significantly to depressive symptoms in persons without MS (20, 21). In line with this finding, Fu et al. (22) showed that individual variations in neurocognitive aversive picture processing can be used to predict the future outcome of depression treatment in persons with major depressive disorder (without MS). Thus, given the close links between stress processing and neuropsychiatric symptoms such as depression on one hand (20–22) and their connection to HRQoL on the other (23), we investigated whether neural activity variations identified in an fMRI stress task can predict the future course of HRQoL in Persons with MS (PwMS). Specifically, we conducted an established fMRI stress task (24, 25) employing mild to moderate stressors (mental arithmetic with social evaluation) to measure neural processes associated with exposure to and cessation of stress at baseline (T0) in 28 PwMS. Conforming with current definitions of psychological relaxation as a process that reduces stress [e.g., (26)], we treated activity variations occurring after stress cessation as neural measures of relaxation. Additionally, we measured pulse and perceived stress levels. We then used the neural markers measured to predict the evolution of HRQoL assessed with the Hamburg Quality of Life Questionnaire in MS [HAQUAMS; (27)] between T0 and a follow-up visit (T1; median delay 902 days). We hypothesized that neural network activity variations triggered by stress exposure and stress cessation predict future HRQoL.

## Materials and Methods

### Participants

This longitudinal study comprising two time points (T0 and T1) is an extension of a study investigating neural stress processing in PwMS and healthy controls at a single time point [i.e., at T0; (28)]. Patients with MS investigated in this recent study were recruited by the Clinical Neuroimmunology Group in the NeuroCure Clinical Research Center (NCRC) in cooperation with the Charité neuroimmunology outpatient clinic. All T0 data of the present longitudinal study was taken from 28 PwMS who participated in a work by Weygandt et al. (28), and for whom HAQUAMS and clinical disability data (gathered in ongoing clinical cohort studies conducted by the NCRC Clinical Neuroimmunology Group) were available for T1. PwMS were included in T0 (i) when diagnosed with relapsing-remitting MS (RRMS) or secondary-progressive (SPMS) MS according to McDonald Criteria 2010 (29), (ii) in case of stable disease-modifying treatment (DMT) for at least 6 months or stable disease without DMT, (iii) when aged ≥ 18 years, and (iv) when physically and mentally capable to use the test devices without restrictions. Candidate participants were excluded when pregnant or when diagnosed with a mental or addictive disorder, neurologic diseases other than MS, acute MS relapses, or acute infections. The exclusion criteria for T1 were the same as for T0 and inclusion criteria (ii) and (iii) were applied in T1. All studies were approved by the research ethics committee of the Charité – Universitätsmedizin Berlin, and written informed consent was obtained from all participants at T0 and T1 according to the Declaration of Helsinki.

Structural MRI and task-derived rating data on perceived stress for T0 and HRQoL data (T0 & T1) were available for all 28 patients (23 RRMS, five SPMS). FMRI data (T0) for two of the three fMRI stages (“Baseline 1” and “Stress”; see below) were available for all 28 participants, and for 27 for the third stage (“Baseline 2”). Pulse data were consistently available across all three fMRI stages for a subset of 21 participants. It is noted that fMRI, structural MRI, pulse, and rating data acquired at T0 were also evaluated in Refs. (19, 28), and T0 HAQUAMS data were also evaluated in Refs. (30, 31).

### Clinical Assessment

Experienced neurologists examined all patients at T0 and T1 using the Expanded Disability Status Scale [EDSS; (32)]. We used the HAQUAMS [Version 3.2, (27)] as an MS-specific, self-report assessment tool for evaluation of overall HRQoL and five (sub-)scales (fatigue [4 items], lower limb mobility [5 items], upper limb mobility [5 items], social functions [6 items], mood-related symptoms [8 items]) at T0 and T1. Compared to other MS-specific HRQoL questionnaires, the HAQUAMS offers the advantage to be both comprehensive including psychosocial factors and disease-specific symptoms as well as feasible regarding completion duration. The total score for overall HRQoL is calculated as the mean across subscales. Patients were neither asked to give their own global rating for QoL nor were they invited to add items other than those listed in the assessment tool used as we aspired to a high degree of comparability between patients. Low scores in each of the five subscales and the total score indicate a high HRQoL. Moreover, the difference in parameters for longitudinal HRQoL variations was computed for each scale (HRQoL at T1 – HRQoL at T0).

### Experimental Stress Paradigm

**Figure 1:**
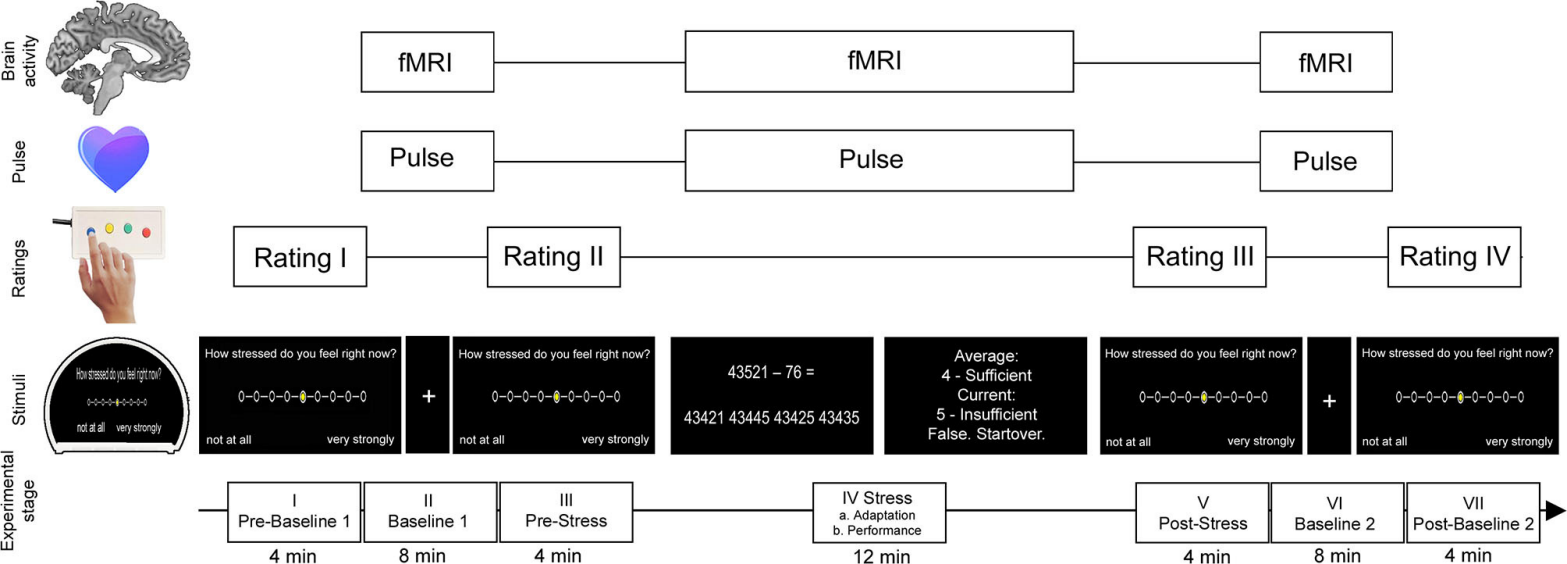
Stress task (for details see “Methods and Materials”). The task consisted of seven stages (I–VII). Neural activity and pulse signals were measured during the first baseline stage (II), the stress stage (IV), and the second baseline stage (VI). The stress stage comprised multiple-choice arithmetic subtraction tasks each followed by feedback depending on individual performance of the participant. During the first part of this stage, the performance level of the participant was assessed (adaption stage) which served as the foundation for the participant's individual level of difficulty for the subsequent performance part of the stress stage. During baseline stages, participants were asked to focus on a fixation cross on the MRI screen. During the prebaseline stage (I), prestress stage (III), poststress stage (V), and postbaseline stage (VII), participants were asked to rate the degree of perceived psychological stress. The figure was adapted from Meyer-Arndt et al. (19).

We applied a version of an established arterial-spin-labeling (ASL) fMRI stress task which was derived from Wang et al. (25) as well as Kirschbaum et al. (33), which was also used in Weygandt et al. (28), and which included mental arithmetic tasks and immediate performance feedback (Figure 1). This task comprised seven consecutive stages (I–VII). During four of these stages (I. prebaseline 1, III. prestress, V. poststress, and VII. postbaseline 2), participants were asked to rate the degree of perceived psychological stress on a nine-point scale, which was displayed on a projection screen inside the MRI scanner using MRI-compatible button tools. The leftmost point corresponded to the option “gar nicht” (German for “not at all”), whereas the rightmost point represented the option “sehr stark” (German for “very strong”). Functional brain activity was acquired using ASL fMRI during the three remaining stages (II. baseline 1; 8 min duration, IV. stress; 12 min, and VI. baseline 2; 8 min). During stages II, IV, and VI, pulse signals were acquired using an MRI-compatible pulse oximeter (refer to Supplement Methods and Materials for details on pulse rate assessment). During baseline 1 and 2, participants were requested to focus on a fixation cross. During the stress stage, participants were asked to perform subtraction tasks (i.e., “operand X minus operand Y”) for which they were to choose the correct result from four options displayed underneath the arithmetical task. The start value for X was set at 43,521 for all participants, whereas operand Y was randomly selected in each trial and ranged from 1 to 99. The stress stage was divided into two parts, an adaptation stage (IVa; ≤ 4-min duration) and a performance stage (IVb; lasting for the remaining time of this block). During the adaption stage, participants were given 8 s per trial to choose a result option. Response times were recorded. In case of a correct result, the difference X minus Y was used as operand X in the next trial. Otherwise, operand X remained the same as in the previous trial. As soon as 10 correct answers were achieved or 4 min had passed, the performance stage began without announcement. The performance stage was different in three aspects. First, the time provided for each trial was adjusted to a given participant's arithmetic performance (starting at 8 s and subsequently decreased or increased by 10% depending on response correctness). Second, feedback was provided in the form of school grades ranging from “1 – sehr gut” (German for “very good”) to “5 – ungenügend” (German for “insufficient”) depending on performance. Finally, X was reset to 43,521 in case of false or too slow answers. Prior to the start of the MRI session, we informed the participants that feedback would relate their output to performance measures established in the general population. After the experiment, we clarified that feedback was actually generated by relating their performance in a given trial in the performance stage to that in the adaptation stage.

### Brain Imaging

As described in Weygandt et al. (28), brain scans at T0 were acquired using a 3 Tesla whole-body tomograph (Magnetom Trio, Siemens, Erlangen, Germany) and a 12-channel head coil. Specifically, fMRI scans were assessed with a pseudo-continuous ASL EPI sequence (25) which roughly covered the whole brain (22 ascending transversal slices, slice thickness 5.75 mm [including 15% inter-slice gap]; in-plane voxel resolution 3 · 3 mm^2^; TR = 4,000 ms; TE = 19 ms; FA = 90°; FOV = 192 · 192 mm^2^; matrix size = 64 · 64; label duration 1.5 s, postlabel delay 1.2 s; phase-encoding direction anterior to posterior). We acquired 120 images (60 control and 60 labels) during baseline 1 and 2 (8-min duration) and 180 scans (90 control, 90 labels) during the stress stage (12 min duration).

Furthermore, two spin-echo EPI reference volumes with opposite phase encoding directions (anterior to posterior, posterior to anterior) were assessed in advance to all three experimental fMRI stages with identical parameters as reported above for a distortion correction of ASL scans described below. We assessed anatomical T1-weighted sequences using the following parameters: 176 slices; slice thickness 1.3 mm; in-plane voxel resolution 1.5 · 1.5 mm^2^; TR = 1,720 ms; TE = 2.34 ms; FA = 9°; FOV = 192 · 192 mm^2^; matrix size = 128 × 128; 1 min and 43 s duration. Additionally, a T2-weighted sequence was acquired with these parameters:176 slices; 1 mm isotropic voxels; TR = 5,000 ms; TE = 502 ms; FA = 120°; FOV = 256 · 256 mm^2^; matrix size = 256 · 256; 5 min and 52 s duration. We opted to use ASL fMRI due to its high sensitivity and its robustness toward slow signal artifacts potentially mimicking stress-induced signal changes and thus impairing proper preprocessing as compared to other functional imaging techniques such as blood-oxygen-level-dependent fMRI (25). We assessed anatomical T1-weighted sequences using the following parameters: 176 slices; slice thickness 1.3 mm; in-plane voxel resolution 1.5 · 1.5 mm^2^; TR = 1,720 ms; TE = 2.34 ms; FA = 9°; FOV = 192 · 192 mm^2^; matrix size = 128 × 128; 1 min and 43 s duration. Additionally, a T2-weighted sequence was acquired with these parameters:176 slices; 1 mm isotropic voxels; TR = 5,000 ms; TE = 502 ms; FA = 120°; FOV = 256 · 256 mm^2^; matrix size = 256 · 256; 5 min and 52 s duration.

### MRI Preprocessing

#### Anatomical Images

Preprocessing of anatomical images comprised of three steps: a manual lesion mapping procedure based on anatomical T2-weighted images, segmentation of brain regions into areas of homogenous tissue based on T1-weighted images, and finally generation of a GM group mask for the predictive fMRI analyses based on segmented images and lesion masks.

#### Lesion Mapping

Experienced raters manually generated patient-specific voxel masks containing focal lesions using the OsiriX software toolbox (OsiriX Foundation) based on T2-weighted images. The procedure was supervised by a neuroradiologist.

#### Segmentation of T1-Weighted Anatomical Images

We used the combined spatial normalization and segmentation SPM12 algorithm to segment the brain of each participant into areas of gray matter (GM), white matter (WM), and cerebrospinal fluid (CSF) and thus to determine voxel images assessing the probability of each coordinate to belong to each of the three tissues based on T1-weighted images. Coordinates highlighted by the manually determined lesion maps coregistered to the T1-weighted images prior to the segmentation step were excluded. Probability maps of GM, WM, and CSF were once computed in the participant-specific (“native”) image space and once in the anatomical standard space defined by the Montreal Neurological Institute [MNI; (34)]. Maps determined in the standard space were adjusted for spatial deformations applied during the normalization. These maps are referred to as “modulated” tissue maps in the following.

#### Computation of a GM Group Mask

In addition to lesion masking and segmentation, we determined a group mask for GM in MNI-space, which was derived from the modulated tissue probability maps of PwMS to constrain our fMRI analyses to functional brain activity signals from GM not affected by lesions as revealed by the procedure described above. Specifically, using the modulated tissue maps, we first determined the voxel-wise average modulated tissue probability for GM, WM, and CSF across all patients. In a second step, we assigned each voxel to the tissue class for which the mean was maximal.

In a third step, we excluded coordinates from the mask containing hyperintense lesions in at least one patient as denoted by the co-registered, patient-specific lesion maps. In Order to account for potential partial voluming effects, we additionally removed the six direct neighbor voxels of each lesion voxels, i.e., coordinates within the Euclidean distance of exactly one voxel to a given lesion coordinate. Finally, we entered voxel coordinates located in the mask and covered by all fMRI scans of all participants into the predictive fMRI analysis.

#### Functional Images

In the present study, we analyzed fMRI data, which were also analyzed in Weygandt et al. (28). In the latter study, seven preprocessing steps were applied. In the present study, data resulting from the first to sixth step were entered into the group analysis, step seven was omitted due to a network-wise approach in the present study (instead of the voxel-wise analysis conducted in Weygandt et al. (28)). Step (ii) was conducted using the FSL Topup algorithm (35), (v) was conducted using the ASLtbx toolbox for SPM12 [Wellcome Trust Centre for Neuroimaging, Institute of Neurology, UCL, London UK - http://www.fil.ion.ucl.ac.uk/spm; (36)]. All other steps were performed using SPM12. Specifically, in step (i) we conducted a coregistration of perfusion fMRI scans to spin-echo EPI volumes to facilitate a distortion correction of functional scans. In step (ii) the distortion correction/B0-unwarping was performed using both spin-echo EPI reference images with opposing phase encoding direction. In (iii) the functional images were coregistered to the T1-weighted images. Moreover, in step (iv) functional scans were spatially smoothed, in (v) voxel maps were determined reflecting the average cerebral blood flow (CBF; ml/100 g/min) across the full 8 min of both baseline stages and the final 8 min of the stress stage based on control-label pairs. It is noted that only the final 8 min of the stress stage were used for computation of neural stress parameters to guarantee equal feedback settings across participants (which may have varied due to individual performance differences or durations of the adaptation stage, respectively, in case of utilizing the full 12 min). In the last step (vi), we used the coregistration parameters determined in the segmentation of T1-weighted scans to map the average CBF maps of the three fMRI conditions to the anatomical standard space defined by the MNI (34). The spatially normalized CBF maps (voxel size of 3 · 3 · 3 mm^3^) resulting from the preprocessing procedure and determined for each participant and all three fMRI conditions separately entered the fMRI group analysis.

### Statistical Analyses

#### Psychophysiological Responses to Stress and Relaxation

To test the effect of stress exposure and its cessation on perceived stress and pulse, we performed linear mixed model (LMM) analyses [e.g., (37)] implemented in Matlab 2014a (MathWorks, Natick, Massachusetts, USA). Specifically, we tested whether stress exposure is accompanied by an increase in perceived stress (stages V vs. III) and pulse (IV vs. II). Moreover, we evaluated whether cessation of stress is accompanied by a reduction of perceived stress (VII vs. V) and pulse (VI vs. IV). The fixed regressor of interest coded zeros (ones) for the earlier (later) stage. In each analysis, MS type of participants (RRMS or SPMS), cognitive task load (an inverse measure of cognitive performance of participants; see Supplementary analysis: “Association between cognitive task load and cognitive performance”), sex, and age (plus intercept) were included in the model as fixed covariates of no interest (CNI) to control for interindividual variability. Finally, an intercept capturing the average stress parameter of each participant across both time points served as random CNI. The false positive rate was evaluated with a permutation strategy for designs with repeated measures [(38); 10,000 permutations]. For analyses of stress exposure, we report parameters with a significant increase, for effects of stress cessation outcomes with a significant decrease (α = 0.05).

#### Longitudinal Variations of HRQoL

To evaluate longitudinal variations of overall HRQoL and HAQUAMS subscales, we also employed LMM. Specifically, a regressor of time coding 0 for T0 and the number of days for the time delay between T0 and T1 for T1 served as a fixed covariate of interest. Participants' sex, age, MS type (RRMS or SPMS), and an intercept served as fixed CNI. Additionally, a random intercept was included. Again, permutation testing was used for inference [(38); 10,000 permutations]. We report HRQoL parameters with significant worsening across time at the group level according to a threshold of α = 0.05. In the Supplement, we additionally investigated whether longitudinal variations in (subscales of) HRQoL were accompanied by similar EDSS variations. It is noted that the linear relationships among HAQUAMS (sub-)scales were computed in the Supplement to yield additional insights into the characteristics of the HRQoL measure in our sample.

#### Predicting Future HRQoL Based on Clinico-Demographic and Radiographic Markers

In this analysis, we tested whether clinico-demographic and radiographic markers assessed at T0 could predict future overall HRQoL. This analysis served two purposes. First, we aimed at testing whether future overall HRQoL could be predicted based on clinico-demographic and radiographic parameters identified as predictors in recent clinical studies [e.g., (18, 39)]. Second, significant clinico-demographic and radiographic markers identified in this analysis were used as additional CNI in the key analysis of the present work described in section Predicting Future HRQoL Based on Neurocognitive Stress and Relaxation Processing. “Predicting future HRQoL based on neurocognitive stress and relaxation processing.”

Specifically, inspired by findings of Baumstarck et al. (18) and Yalachkov et al. (39), we tested the prognostic information of nine clinico-demographic and radiographic markers for future overall HRQoL (i.e., the difference in the total HAQUAMS scores for T1 minus T0) in nine separate robust regression analyses (one for each marker). The nine markers evaluated were: sex, age, education, clinical disability, the annualized relapse rate, T2-weighted lesion load, MS type (RRMS or SPMS), disease duration, and overall GM fraction of participants. Robust regression was used due to its statistical power and reduced sensitivity to outliers compared to traditional ordinary least square regression (40–42). Total HAQUAMS scores at T0 and the duration of the interval between T0 and T1 in days (plus constant) were entered in each of the nine analyses as CNI. A robust permutation method proposed by DiCiccio and Romano (41) relying on the Chi^2^-distributed Wald-statistic was used to compute the false positive rate (10,000 permutations in each analysis). Parameters were considered significant predictors of longitudinal HRQoL if the false positive rate determined for two-sided effects α was < 0.05.

#### Predicting Future HRQoL Based on Neurocognitive Stress and Relaxation Processing

In this analysis, we investigated whether activity changes of neural networks triggered by stress exposure or by its cessation predict the future evolution of HRQoL. We applied a technique named principal component regression [e.g., (43)], which combines two methods widely used in neuroimaging research, i.e., principal component analysis [PCA; (44, 45)] and linear regression (46). The analysis is comprised of three major steps.

In the first, we identified neural networks composed of areas whose activity was strongly characterized by the activity of a given network during the stress stage. Specifically, we first determined the average voxel CBF of each participant and each GM region included in the Neuromorphometrics neuroanatomical atlas (http://Neuromorphometrics.com) for each fMRI stage (i.e., covering the full 8 min of both baseline stages and the final 8 min of the fMRI stress stage) individually. Next, we centered regional mean CBF signals of the participants by subtracting their average overall GM CBF (i.e., the average computed across all average GM region CBF signals) for each fMRI stage individually. Subsequently, we employed PCA to determine a small number of hidden or latent variables (i.e., principal components; PCs) based on the centered regional GM CBF signal of the fMRI stress stage [compare e.g., (47) on the use of averaged regional fMRI signals in functional network studies]. The PCs (which are similar to “factors” in factor analyses of questionnaire data) reflect the shared characteristic variation underlying the signals of individual regions contributing to a given network during the stress stage across participants. Each individual PC represents one network and encodes the network activity of a participant in terms of a single number. The number of PCs is predetermined by the input data and corresponded to 28 (i.e., the number of participants) in this study.

In the second step, we computed differential network activity parameters reflecting the effect of stress exposure and its cessation. Specifically, we first used the centered regional activity signals of both baseline stages to determine the activity of the network during these stages. Afterward, we subtracted the network activity scores for baseline 1 from those for stress (stress exposure) and the activity scores for stress from those for baseline 2 (cessation of stress). Refer to the Supplement for further information (including on the “Winner-Takes-All” method used to determine which brain regions are related to which networks).

In the third step, we used the differential network activity parameters to predict the longitudinal evolution of overall HRQoL with robust regression. This was done for each neural process type (i.e., stress exposure or cessation of stress), and each of the 28 networks. Complementary analyses tested these associations for all five HAQUAMS subscales. To account for the potential impact of confounding variables, we included generic nuisance factors in the robust regression model (to address basic longitudinal modeling or cognitive neuroscience aspects) as well the disease-specific variable(s) identified in preparatory analysis 2.6.3 (to address for clinico-demographic aspects). Given that only disease type (SPMS vs. RRMS) but none of the other eight markers including education and clinical disability was significantly associated with future overall HRQoL variations according to the analysis described in 2.6.3 (see Results section Predicting Future HRQoL Based on Clinic-Demographic and Radiographic Markers), we included three generic CNI (follow-up period duration per participant in days, T0-HAQUAMS scores in a given scale, and cognitive task load) and one disease-specific (disease type). It is noted that this sequential analysis scheme (i.e., identification of significant clinico-demographic or neuroradiographic variables in preparatory analysis, section Predicting Future HRQoL Based on Clinico-Demographic and Radiographic Markers, and inclusion of identified significant predictor variables in key analysis, section Predicting Future HRQoL Based on Neurocognitive Stress and Relaxation Processing) was applied to avoid an unnecessary reduction in statistical power which would have followed from including all nine (mostly HRQoL-unrelated) clinico-demographic or neuroradiographic variables. We deployed the same robust permutation method proposed by DiCiccio et al. (41) as in the previous analysis. For the key outcome markers (future variation in overall HRQoL), we report significant associations according to multiple comparisons or FWE corrected threshold for two-sided effects of α_FWE_ = 0.05, which was computed with the Bonferroni method (i.e., by dividing the false positive rate of a single test [α = 0.05] by the number of PCs [*N* = 28]). Within this framework, the uncorrected equivalent of α_FWE_ = 0.05 was α_uncorrected_ = 0.0018. For the complementary (i.e., subscale) analyses, we applied a threshold of α_FWE_ = 0.1. The uncorrected equivalent of α_FWE_ = 0.1 was α_uncorrected_ = 0.0036.

## Results

### Demographic and Clinical Participant Characteristics

A total of 28 PwMS (23 RRMS, five SPMS) participated in this study. Eighteen participants were women and 18 participants obtained at least a high school diploma. The median age at T0 was 49 years (range: 27–61) and at T1 51 years (range: 29–64). The median disease duration (since the first manifestation) at T0 was 3,491 days (range: 271–12,250). Moreover, the median EDSS was 3.5 (range: 1–6) at T0 and 3 (range: 1–6) at T1. The median time between T0 and T1 was 902 days (range: 363–1,169). At T0, 20 patients received DMT (six interferon beta, six glatiramer acetate, five dimethyl fumarate, three fingolimod), at T1 21 PwMS received DMT (three interferon beta, six glatiramer acetate, six dimethyl fumarate, four fingolimod, one teriflunomide, one ocrelizumab).

### Psychophysiological Stress and Relaxation Responses

**Figure 2:**
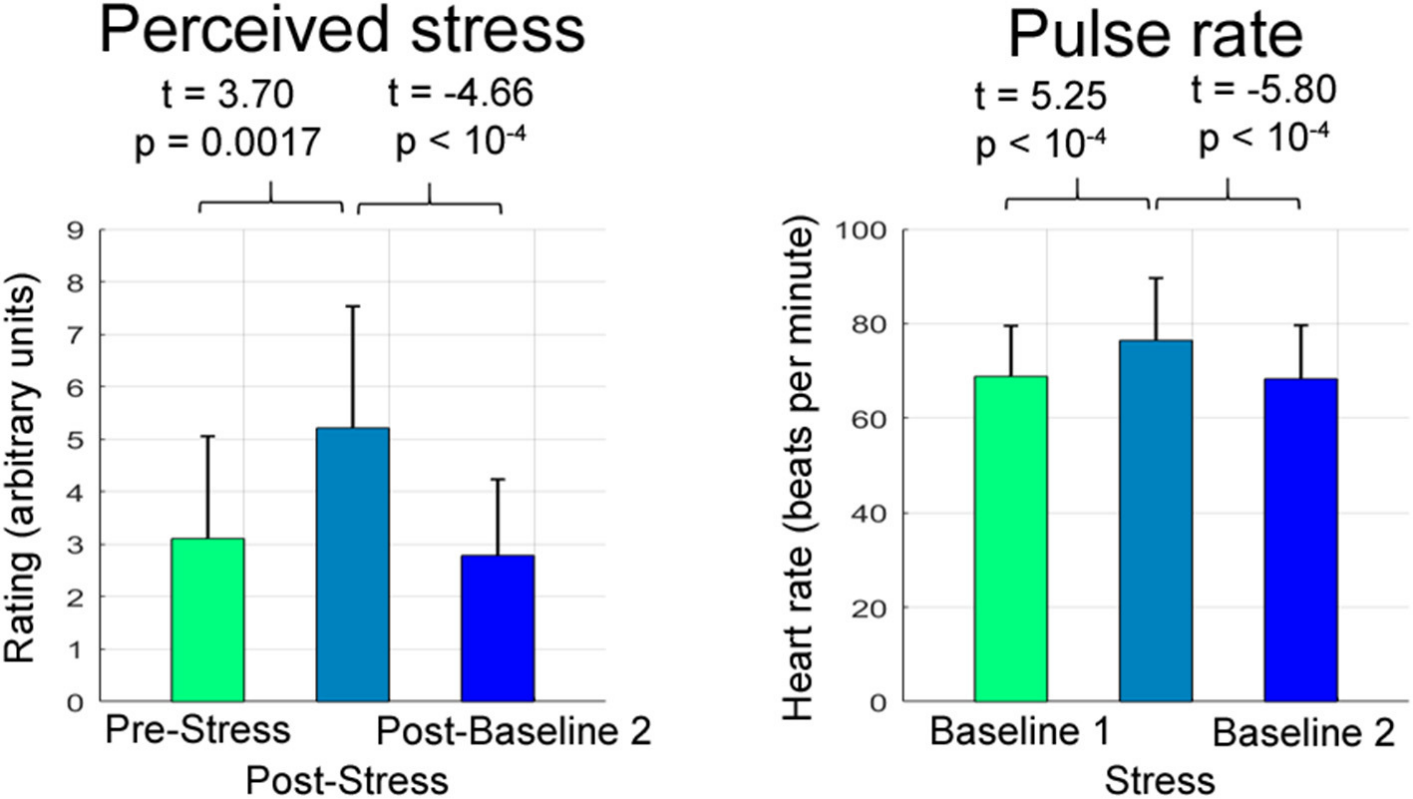
Psychophysiological stress and relaxation responses. The bar graphs depict the mean and the standard deviation of raw perceived stress ratings and pulse rates (i.e., ratings and pulse rates not corrected for CNI) across participants separately for the respective experimental stage.

Stress exposure triggered a pronounced increase in pulse (*t* = 5.25, *p* < 10^−4^) and perceived stress (*t* = 3.70, *p* = 0.0017), cessation of stress induced a substantial decrease in both parameters (pulse: *t* = −5.80, *p* < 10^−4^; perceived stress, *t* = −4.66, *p* < 10^−4^; Figure 2).

### Longitudinal Variations of HRQoL

**Figure 3:**
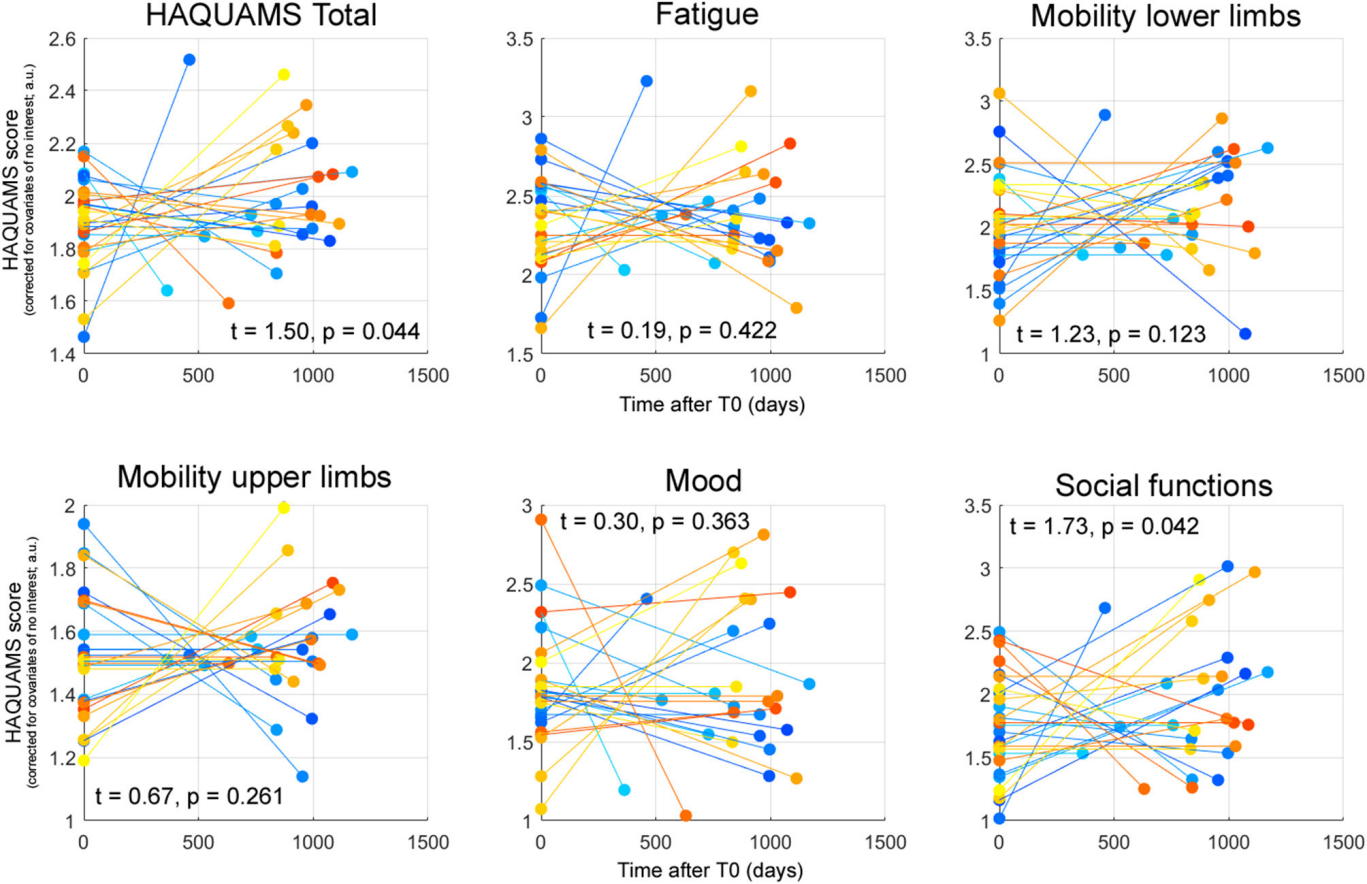
Patient-specific HAQUAMS scores for overall HRQoL and five HAQUAMS subscales at T0 and T1 corrected for the CNI mentioned in the text. Each line depicts the trajectory of the given marker for an individual participant. The *t*-statistics and *p*-values reported characterizing the effect of time on each given parameter across all 28 PwMS. It is noted that higher scores correspond to less HRQoL, positive *t*-statistics to a (not necessarily significant) increase of a marker across time (and thus to a not necessarily significant HRQoL worsening).

Table 1 depicts the raw scores for the total HAQUAMS score and the five subscales. The temporal variations of each parameter for each PwMS and corresponding parameters of inferential statistics, indicating a significant worsening of overall HRQoL and social functioning, are shown in Figure 3.

**Table 1:** Depicts raw scores (i.e., not corrected for CNI) for all six HAQUAMS parameters and both time points.

	**Total**	**Fatigue**	**Mobility lower limbs**	**Mobility upper limbs**	**Mood**	**Social functions**
T0	1.77 1.25–2.97	2.25 1.00–3.75	1.70 1.00–4.20	1.20 1.00–3.60	1.69 1.25–3.00	1.63 1.00–3.00
T1	1.94 1.00–3.04	2.13 1.00–4.00	2.20 1.00–4.20	1.40 1.00–3.20	1.69 1.00–3.38	1.75 1.00–4.17

*Lower scores indicate a higher HRQoL. Specifically, the number reported in the upper line of each cell reports the median and the numbers in the lower line report parameter range across all 28 PwMS*.

### Predicting Future HRQoL Based on Clinic-Demographic and Radiographic Markers

**Figure 4:**
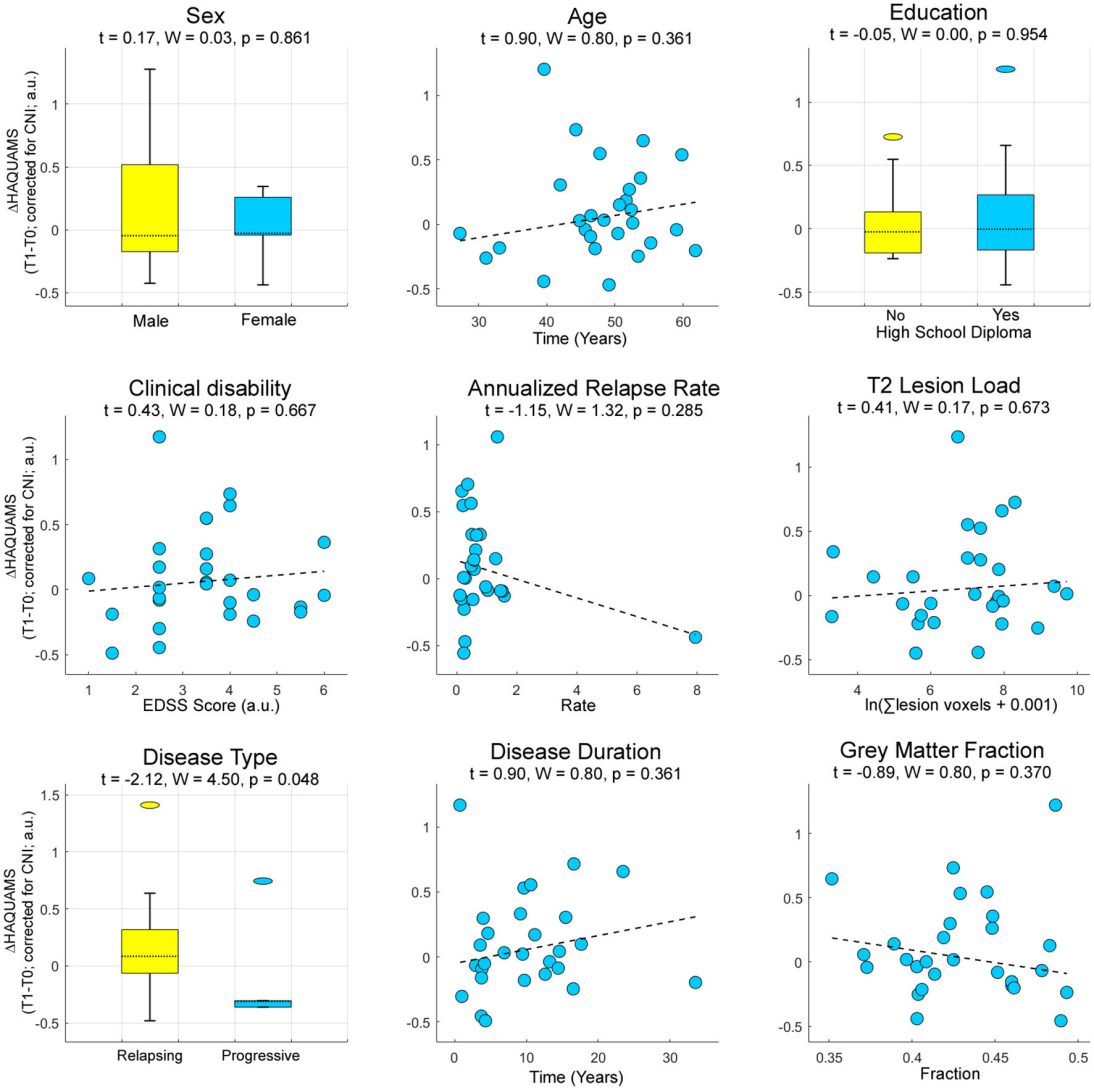
Prognostic information of established clinical, demographic, and radiographic parameters for future variations in overall HRQoL. Higher ΔHAQUAMS scores correspond to stronger longitudinal declines in HRQoL. W, Wald-statistic.

To predict future HRQoL based on clinic-demographic and radiographic markers, we tested for sex, age, education, clinical disability, annualized relapse rate, T2-weighted lesion load, MS type (RRMS or SPMS), disease duration, and overall GM fraction as potential sources of interindividual variation of the participants. Among these, only disease type (RRMS or SPMS) was predictive of future variations in the total HAQUAMS score (Figure 4).

### Predicting Future HRQoL Based on Neurocognitive Stress and Relaxation Processing

**Figure 5:**
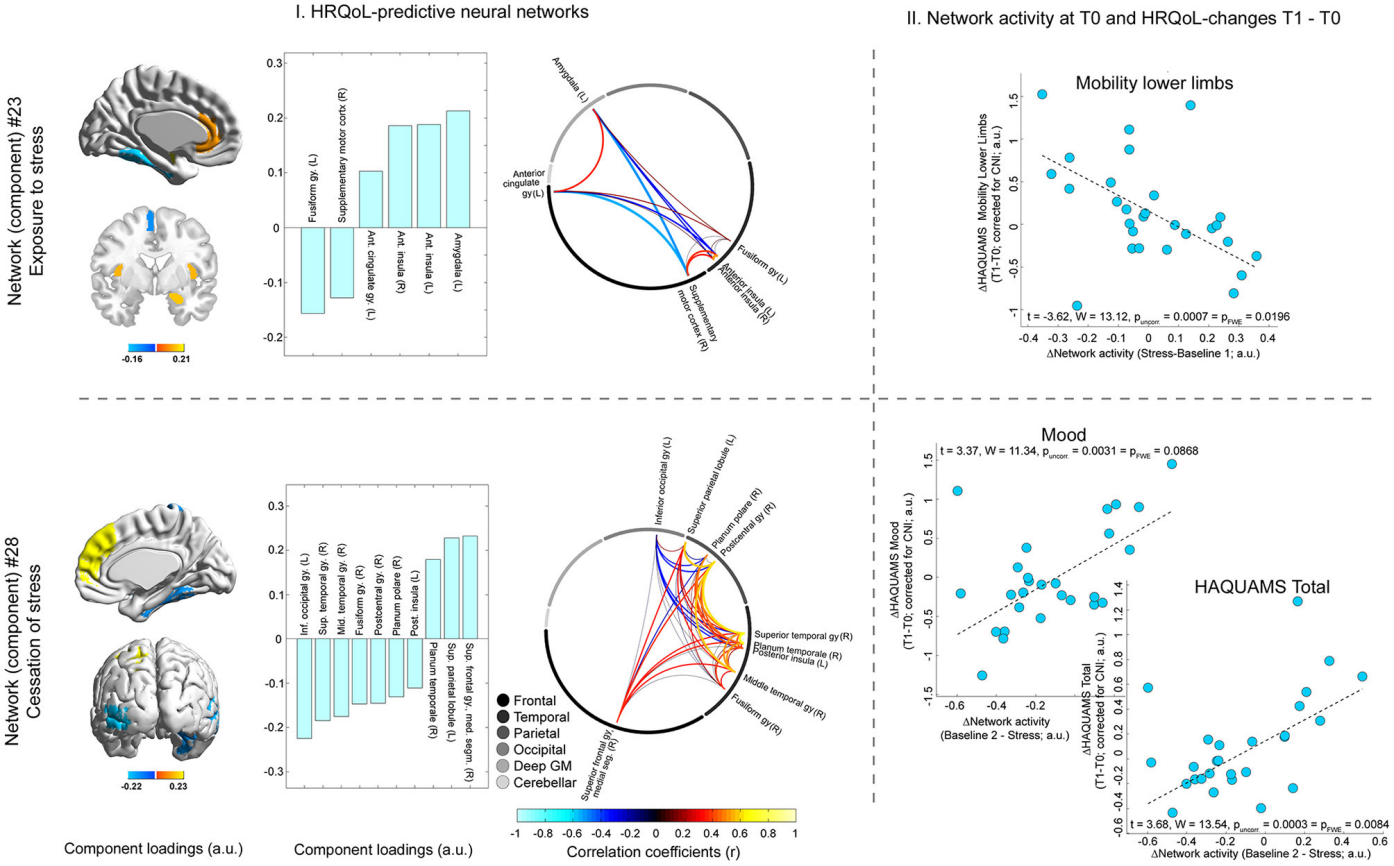
Neural network activity and future HRQoL evolution. T0 activity of one network (network #23, shown in the upper row of the figure) triggered by stress exposure (which was reflected by the difference in network activity for stress minus baseline I) was predictive of future variations in lower limb mobility. The activity of another network (#28, shown in the lower row of the figure) triggered by cessation of stress (reflected by the differential network activity for baseline II minus stress) was predictive of future variations in overall HRQoL (i.e., total HAQUAMS score) and mood. The left side of the figure (“I. HRQoL-predictive neural networks”) depicts different features of the two networks. In particular, the rendered brains in the leftmost column of (I.) and the bar graphs to their right depict individual brain regions included in the neuroanatomical atlas related to the predictive networks and the strength and direction of this relation (i.e., their component loadings; see Supplement for details on the computation of these loadings as well as a comprehensive overview of loadings of all atlas regions). The circular network graphs on the rightmost side of (I.) illustrate the intercorrelations of manifest regional signals for areas belonging to a given network. It is noted that the strength and direction of these intercorrelations do not only depend on the fact that the given regions are all maximally related to the same network but also on weaker relations to other networks and that the contribution of these other networks to the activity of regions may vary across regions. The scatter graphs on the right side of the figure (“II. Network activity at T0 and HRQoL-changes T1–T0”) illustrates the associations between a given differential network activity marker (i.e., for stress or relaxation) and the temporal difference score of the given HAQUAMS parameter. Positive differences ΔHAQUAMS denote worsening in HRQoL ratings over time.

This analysis identified one neural network comprising (para-)limbic regions (i.e., anterior insula, left amygdala, and left anterior cingulate cortex and also left fusiform gyrus and right supplementary motor cortex) whose stress-triggered activity variations were positively associated with future lower limb mobility (*t* = −3.62, *p*_FWE_-corrected = 0.020; Figure 5). Higher activity variations during stress cessation of a different widespread network predicted lower overall HRQoL (*t* = 3.68, *p*_FWE_ = 0.008) and mood on trend level (*t* = 3.37, *p*_FWE_ = 0.087; Figure 5). This second network included the following areas: right superior frontal gyrus (medial segment), left superior parietal lobule, right planum temporale, and also left inferior occipital gyrus, right superior and middle temporal gyrus, right fusiform gyrus, right postcentral gyrus, right planum polare, and left posterior insula (Figure 5).

## Discussion

We investigated whether brain activity triggered by mild psychological stress or by its cessation predicts the future evolution of HRQoL in 28 PwMS.

In a first analysis, we showed that the fMRI stress task employed induced a stress response increasing perceived stress and pulse and that stress cessation was accompanied by the remission of both parameters. Together, these findings emphasize the fundamental ability of our task to induce a psychophysiological stress response and measure relaxation after stress.

In a second analysis, we characterized the evolution of HRQoL across the follow-up period and revealed that overall HRQoL and social functioning deteriorated significantly, a finding which is compatible with an inverse association of MS disease duration and future HRQoL (18). Furthermore, a supplementary analysis testing whether longitudinal HRQoL variations were accompanied by similar changes in EDSS showed a pronounced positive association for the HAQUAMS lower limb mobility subscale and EDSS (and between EDSS and the total HAQUAMS score on an α = 0.1 level). The associations between EDSS and other HAQUAMS subscales were much less pronounced. This finding underlines the importance of HRQoL as a complementary factor in the assessment of MS and patient-reported outcomes for treatment success.

Finally, in the key analysis of the study, we tested whether neural network activity changes induced by exposure to or cessation of stress (i.e., relaxation) can be harnessed to predict the future evolution in overall HRQoL. Complementary analyses tested the same effects for the HAQUAMS subscales. The key analysis showed that the activity of a widely distributed network triggered by cessation of stress (i.e., relaxation) was predictive of overall HRQoL aggravation, and the complementary analyses showed that activity of the same network was predictive of mood-related HRQoL aggravation on an α_FWE_ = 0.1 level. Specifically, the weaker the activity decline of this network after stress (i.e., the less neural relaxation), the more pronounced the reduction in overall and mood-related HRQoL. The brain regions identified as contributing to this network could provide a clue to the nature of the positive link between neural relaxation and mood-related HRQoL from a cognitive neuroscience perspective. In particular, the network comprised the medial segment of the right superior frontal gyrus, which is located in the dorsomedial PFC (dmPFC). Neuroimaging studies found that dmPFC activity is related to self-referential processing during emotion regulation (48). Moreover, depressed patients show greater activity in this area during emotion regulation tasks than healthy controls (49), and stronger dmPFC activity during such tasks is positively linked to the severity of future depressive symptoms (50). Thus, given the positive association between the activity of this network and the right superior frontal gyrus (bar graph lower panel Figure 5), this finding could suggest that protracted self-reference after cessation of stress is a factor related to future mood-related HRQoL aggravation in MS. This interpretation of an altered relaxation processing in MS would also be consistent with the results of our recent study showing that PwMS have difficulty in integrating peripheral stress signals into the perception of relaxation (51). Moreover, when additionally considering the high correlation between temporal differences in mood-related and overall HRQoL of *r* = 0.80 (which was the highest correlation among all pairs of [sub-] scales; refer to Supplementary Figure 2, right panel), these arguments might legitimately also be used to explain the link between the activity of this network and future aggravation in overall HRQoL because overall HRQoL assessed by HAQUAMS is particularly sensitive to mood-related HRQoL.

In addition to these findings on associations between neural relaxation and HRQoL, a complementary analysis showed that stress-induced activity of a network comprising prefrontal regions (i.e., anterior cingulate cortex [ACC], an area located in the ventromedial prefrontal cortex; vmPFC), limbic (amygdala), and paralimbic regions (anterior insula) was negatively linked to future worsening of lower limb mobility scores. In other words, the stronger the network's stress response, the lower the self-reported future aggravation of lower limb mobility. Two potential explanations come into mind for this association. First, in line with findings of our recent longitudinal study showing that stress-induced activity of a prefronto-limbic network predicts the future atrophy of cerebellar areas in PwMS (19), one might assume that central stress processing directly contributes to brain atrophy of mobility-related regions and thus to subsequent motor impairment. Neuropathological candidate mechanisms that could mediate this association were documented in animal work, showing that sustained experimental stress exposition (52) and sustained excessive glucocorticoid release (53) can provoke loss of dendritic spines. This explanation would also be in line with our recent crosssectional findings showing that stronger anterior insula stress responses are accompanied by less severe pyramidal symptoms in PwMS (28), with vmPFC-mediated glucocorticoid regulation found in healthy persons by Urry et al. (54), and with findings showing that stronger cortisol awakening responses in PwMS are associated with worse EDSS 9 months later (16). Second, associations between central stress processing and perceived future aggravation of lower limb mobility might be explained by functional processes also observed in motor functional neurological disorders. In particular, these disorders are frequently accompanied by psychological stress (55), are characterized by limb weakness or paralysis, functional movement disorders (55), alterations in resting state functional connectivity (56), and an absence of obvious structural brain damage (55). This explanation might also be compatible with associations between resting-state functional connectivity and future disease worsening across a time span of 6.4 years in PwMS observed by Rocca et al. (57). Importantly, the first and the second explanation must not necessarily be mutually exclusive. Instead, one might speculate that impairment in (perceived) lower limb mobility in MS is mediated by a mixture of functional and structural factors.

Some limitations of the present work should be mentioned. One limitation is the lack of a control group, which impedes evaluation of whether the observed associations are specific to MS. However, as this study is the first to address neural predictors of future HRQoL in MS, we consider the predictors identified as an important foundation for further research. Another aspect that should be mentioned is the only moderate sample size of this task-based fMRI MS study. Thus, future studies addressing associations between neural correlates of psychological stress (or of other factors with neuropsychiatric importance) on one hand and QoL on the other should rely on a larger number of participants to facilitate analyses with higher statistical power. However, the fact that the brain areas contributing to HRQoL-predictive networks identified in this study (e.g., dmPFC, amygdala, vmPFC, anterior insula) are well in line with those found in other studies investigating stress or stress-related factors in MS (28, 51) or independent of MS (48–50, 54) suggests that the statistical power of our analyses does not fundamentally question the results obtained. Another possible limitation is confounding variables, such as Vitamin D (58), the gut microbiome (59), physical exercise (60), or sleep disturbances (61), which pose a challenge to any observational MS study given the heterogeneous set of factors considered to influence the disease. At this point, we want to mention, however, that a significant number of conceivable factors were considered in the key robust regression fMRI analysis on future HRQoL prediction as CNI. Specifically, we accounted for the T0 HAQUAMS scores of a given subscale, the follow-up period (i.e., time between T0 and T1), and cognitive task load (which was shown to be an inverse measure of participants' cognitive performance). Additionally, we tested for possible clinico-demographic and radiographic predictors of future HRQoL variations and consequently included disease type (SPMS vs. RRMS) as CNI in the respective regression models. None of the other markers tested (such as clinical disability or education) were predictive of future changes in HRQoL. Finally, depression was not modeled as CNI because one of the predicted HAQUAMS subscales (“mood”) is itself considered as a measure of depression (27). Accounting for this factor by including alternative measures would thus presumably have removed the target variation. Consequently, we assume that the impact of putative nuisance factors was not very relevant. Patients with primary progressive MS were not included in the study as this entity has often been considered diverging from other clinical MS courses (62, 63). Future studies evaluating the prognostic potential of stress- and relaxation-related brain activity should consider further socio-demographic variables as confounding factors as Baumstarck et al. (18) showed that occupational and marital status are related to HRQoL. A final aspect to be discussed is that in this work only PCs with a high rank showed specific associations to MS severity measures. This may find fault with other authors who propose that only PCs of low rank (i.e., explaining a lot of variation) can be meaningful for PC regression [e.g., (64)]. However, this heuristic was already refuted by Jolliffe (43).

In conclusion, we showed that variations in neural network activity triggered by stress exposure and its cessation can predict the future course of HRQoL in PwMS. Our findings underline the relevance of unimpaired stress processing and poststress relaxation for psychobiological well-being and thus advocate a strengthening of stress coping skills in the treatment of MS.

## Data Availability Statement

The raw data supporting the conclusions of this article will be made available by the authors, without undue reservation.

## Ethics Statement

The studies involving human participants were reviewed and approved by Ethics Committee of the Charité—Universitätsmedizin Berlin. The patients/participants provided their written informed consent to participate in this study.

## Author Contributions

MW, FP, and SG: conceptualization and funding acquisition. MW and LM-A: data analysis and writing. MW, LM-A, TS-H, AB, JB-S, and J-DH: resources. All authors contributed to the article and approved the submitted version.

## Funding

This work was supported by the German Research Foundation (WE 5967/2-1 to MW, GO1357/5-2 and GO1357/9-1 to SG, and Exc 257 to FP). Our funding sources did not influence the study design, the collection, analysis and interpretation of data, the writing of the report, or the decision to submit the article for publication.

## Conflict of Interest

The authors declare that the research was conducted in the absence of any commercial or financial relationships that could be construed as a potential conflict of interest.

## Publisher's Note

All claims expressed in this article are solely those of the authors and do not necessarily represent those of their affiliated organizations, or those of the publisher, the editors and the reviewers. Any product that may be evaluated in this article, or claim that may be made by its manufacturer, is not guaranteed or endorsed by the publisher.
