# Supplementary material for: Neural Processes of Psychological Stress and Relaxation Predict the Future Evolution of Quality of Life in Multiple Sclerosis
Source: Front Neurol. 2021 Nov 23;12:753107. doi: 10.3389/fneur.2021.753107 (PMC8650716; doi:10.3389/fneur.2021.753107)
Supplement: Supplementary file 1 [file Data_Sheet_1.docx]

Supplementary Material

# Supplementary Data

## Methods and Material

### Heart rate acquisition

We used a standard pulse oximeter included in the Physiological Monitoring Unit (PMU) of the MRI scanner to measure peripheral pulse during all three fMRI stages, i.e., Baseline 1, Stress and Baseline 2 (sampling frequency 50 Hz). During measurement, the pulse oximeter was placed on the participants’ toe to avoid potential transmitter dislocation by button presses during the task. We excluded values of pulse acceleration up to 133% and decline to less than 75% for denoising. Moreover, as for the processing of fMRI data, we included only pulse signals acquired in the last 8 minutes of the Stress stage.

### Determination of cognitive task load and cognitive performance

We computed cognitive task load as the average time between arithmetic trials across the final eight minutes of the Stress stage and included this variable in predictive fMRI analysis as CNI. Due to the variability of the time between mental arithmetic trials during this experimental period depending on participants’ performance (10% less time after correct trials, 10% more time after false trials), cognitive task load was an inverse measure of cognitive performance. To demonstrate this negative association, we also computed the number of correctly solved trials during the final eight minutes of the Stress stage. Please see also *Supplementary analysis: Association between cognitive task load and cognitive performance* below.

### Predicting future HRQoL based on neurocognitive stress and relaxation processing (determination of neural network activity variations in response to stress and relaxation)

In the main analysis of the present study, we investigated whether activity changes of neural networks in response to stress (i.e., for Stress relative to Baseline 1) and relaxation (i.e., for Baseline 2 relative to Stress) predicted the longitudinal evolution of HRQoL and its facets. In the following, we describe the computation of the neural network activity measures. In a first step, we mathematically identified neural networks based on regional brain activity during the Stress stage using Singular Value Decomposition (SVD). SVD is a firmly established signal processing technique frequently employed for the analysis of extensive multivariate biological data (Mourao-Miranda et al., 2005; Wall et al., 2003). In particular, this technique decomposes a given set of directly observable (i.e., ‘manifest’) data coded in matrix **X** into three matrices **U**, **S** and **V**. In this work, the elements of **X** contained the average CBF for each patient (one patient per matrix row) and 121 regions (one region per matrix column) comprised in the Neuromorphometrics atlas and covered by the GM group mask. More specifically, matrix **X**_Baseline 1_ contained these fMRI data for the Pre-Stress, matrix **X**_Stress_ the data for the Stress, and **X**_Baseline 2_ the data for the Post-Stress stage. After calculation of the average regional CBF values for **X**_Baseline 1_, **X**_Stress_, **X**_Baseline 2_, we standardized (i.e., centered) these measures by subtracting the average CBF of a given patient across all 121 regions during a given experimental stage. We then applied SVD to **X**_Stress_ and obtained **U**_Stress_, **S**_Stress_ and **V**_Stress_. Columns of **U**_Stress_ contained the PCs of **X**_Stress_. These variables, which are not directly observable (i.e., ‘latent’) reflect the activity of a given network (one column per network) across participants (one column element in a column per patient) characterizing the activity of individual regions (columns in **X**_Stress_) related to this network. How strongly a given region is related to a network depends on network-specific columns of **V**_Stress_, a matrix containing the region-wise component loadings (e.g., Wall et al., 2003). (See also the next paragraph *Defining which brain regions are related to which neural networks).* **S**_Stress_ is the matrix of singular values coding the impact/magnitude with which a corresponding PC contributes to the manifest activity signals of the individual regions. Finally, reconstruction of the raw data (i.e., the centered regional CBF signals) is possible by computing **X** = **U** ∙ **S** ∙ **V**^T^. Given that we were interested in differential network responses reflecting the impact of Stress relative to Baseline 1 and of Baseline 2 relative to Stress, we then determined the activity of our networks (which were inferred from the Stress stage) during Baseline 1 and 2 by computing **U**^*^_Baseline 1_ = **X** _Baseline 1_ ∙ (**S**_Stress_ ∙ **V**^T^_Stress_)^+^ and **U**^*^_Baseline 2_ = **X** _Baseline 2_ ∙ (**S**_Stress_ ∙ **V**^T^_Stress_)^+^. In this case, the ^+^ denotes the Moore-Penrose pseudoinverse of a matrix. Finally, the matrices **U**^*^_Baseline 1_ and **U**^*^_Baseline 2_ were used to compute the differential network response matrices **∆U**_Stress_ = **U**_Stress_ – **U**^*^_Baseline 1_ and **∆U**_Relaxation_ = **U**^*^_Baseline 2_ – **U**_Stress_. Please note that it was not feasible to determine the differential network responses by simply calculating e.g., **∆U**_Stress_ = **U**_Stress_ – **U**_Baseline 1_ (i.e. using network activity **U**_Baseline 1_ as determined by SVD of **X**_Baseline 1_) as the PCs identified by SVD rely on the covariation or connectivity between regions and this covariation differs across fMRI conditions. Consequently, the PCs (i.e., neural networks) identified by SVD of **X**_Baseline 1_ would have been different from those found by SVD of **X**_Stress_. Supplementary Figure 1 illustrates this network activity computation procedure.

### Defining which brain regions are related to which neural networks

Following a “Winner-Takes-All” strategy, we considered a region as part of a given network/component if the relative contribution of the component’s signal to the regional signal (assessed in terms of its absolute value in **V**) was larger than that of any other component.

### Statistical analyses

#### Association between cognitive task load and cognitive performance

### To demonstrate that cognitive task load is an inverse measure of cognitive performance (this necessarily follows from the adaptive trial time adaptation scheme implemented in our stress task), we computed the Pearson product moment correlation coefficient between both factors.

#### Correlation among HAQUAMS (sub-) scales

To assess the linear relationships among HAQUAMS (sub-) scales, we computed the Pearson correlation coefficient among the all six (sub-) scales separately for T0, T1, and for the (sub-) scale differences across time (i.e., for T1 minus T0).

#### 1.1.5.3 Longitudinal evolution of HRQoL (relation to the EDSS)

Here, we tested whether longitudinal variations of the HAQUAMS total score or its five subscales were accompanied by similar longitudinal variations in EDSS in six separate LMM analyses (one for each HAQUAMS parameter). In each analysis, a regressor coding the participants’ EDSS scores for T0 and T1 served as covariate of interest. Regressors of time coding 0 for T0 and the number of days for the timely delay between T0 and T1, the participants’ sex, age, MS type (RRMS or SPMS) and an intercept served as fixed covariates of no interest. The T0 and T1 scores of a given HAQUAMS (sub)scale served as dependent variable. Finally, an intercept capturing each participant’s average HAQUAMS total score across both time points served as random covariate of no interest. We used permutation testing using the strategy proposed by Winkler et al. (2014) for designs with repeated measures (10,000 permutations) to test for positive associations between EDSS and HAQUAMS parameters.

## Supplementary Results

### Association between cognitive task load and cognitive performance

To underline the close negative association between task load and task performance (which necessarily followed from the adaptive trial time adaptation scheme implemented in our stress task), we computed the Pearson product moment correlation coefficient, which revealed r = -0.93, p < 10^-4^.

### Correlation among HAQUAMS (sub-) scales

The Person correlation coefficients computed for linear relationships among HAQUAMS (sub-) scales are depicted in Supplementary Figure 2.

### Longitudinal evolution of HRQoL (relation to the EDSS)

This analysis revealed a pronounced positive longitudinal association between EDSS and the HAQUAMS subscale for lower limb mobility (t = 4.28, p < 10^-4^). Please note that this significant positive association is possible despite the fact that the median values of both markers varied slightly in opposite direction across time as both markers showed a comparably strong positive intra-time point association. Moreover, for the positive association of EDSS and the total HAQUAMS score a t-statistic of t = 1.39 (p = 0.086) was determined. The remaining positive associations were much less pronounced (fatigue: t = 0.23, p = 0.116; upper limb mobility: t = 1.02, p = 0.157; mood: t = -0.20, p = 0.421; social functions: t = 0.49, p = 0.314).

## Supplementary References

Mourao-Miranda J, Bokde AL, Born C, Hampel H, Stetter M (2005). Classifying brain states and determining the discriminating activation patterns: Support Vector Machine on functional MRI data. NeuroImage 28: 980-95.

Wall ME, Rechtsteiner A, Rocha LM. Singular value decomposition and principal component analysis. In A Practical Approach to Microarray Data Analysis (D.P. Berrar, W. Dubitzky, M. Granzow, eds.) Kluwer: Norwell, MA, 2003. pp. 91-109.

Winkler AM, Ridgway GR, Webster MA, Smith SM, Nichols TE (2014). Permutation inference for the general linear model. NeuroImage 92: 381-97.

# Supplementary Figures

**
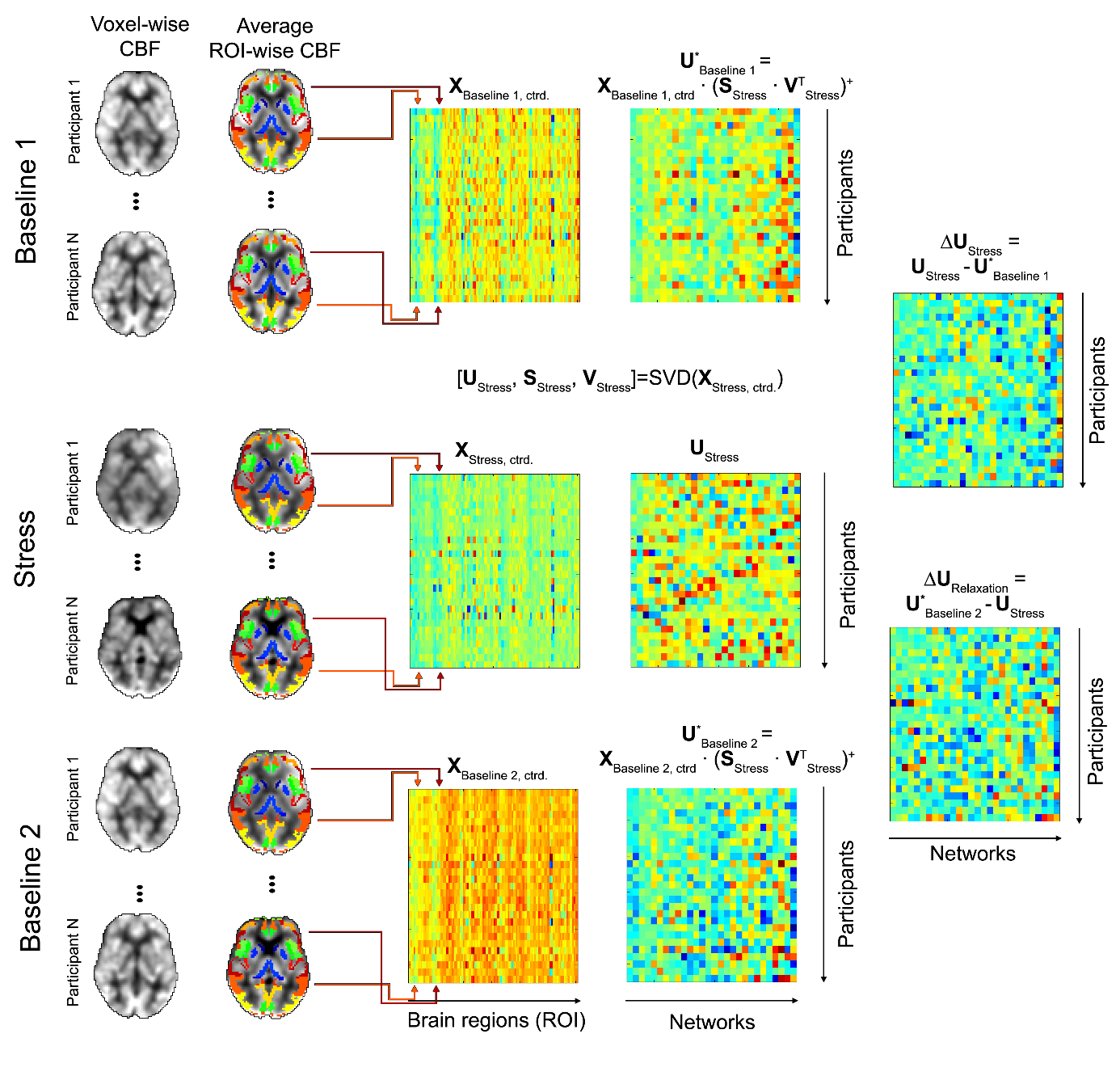
Supplementary Figure 1.** Computation of network activity parameters. Abbrev.: ctrd., centered. For details see text.


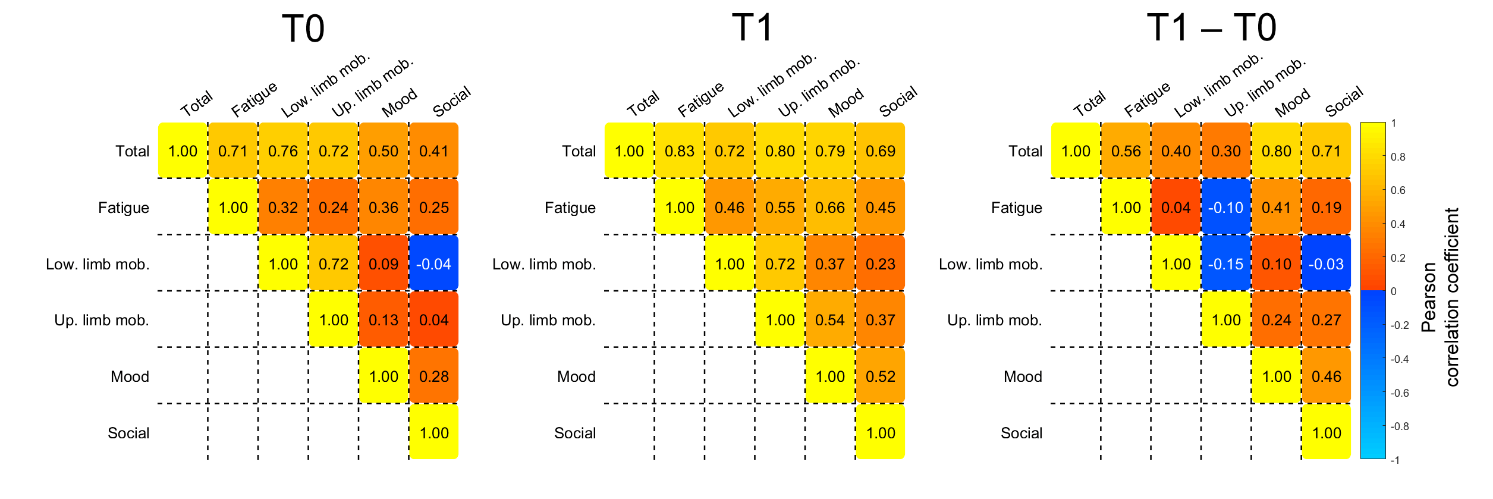
 **Supplementary Figure 2** depicts the Pearson correlation coefficients separately computed for all six HAQUAMS (sub-) scales (from left to right) for T0, T1, and for the (sub-) scale differences across time (i.e., for T1 minus T0). Abbreviations: Low., lower, mob., mobility, Up., upper.
